# Randomized Clinical Trial on the Efficacy of Oral Tranexamic Acid Versus Topical Tranexamic Acid in Treatment of Melasma

**DOI:** 10.1111/jocd.70428

**Published:** 2025-09-09

**Authors:** Behrooz Heidary, Golnaz Alsadat Habibzadeh Meibodi, Mohammad Ebrahimzadeh Ardakani, Vahid Ramezani, Fateme Heidari

**Affiliations:** ^1^ Department of Pharmaceutics, Faculty of Pharmacy Shahid Sadoughi University of Medical Sciences Yazd Iran; ^2^ Department of Dermatology Shahid Sadoughi University of Medical Sciences Yazd Iran; ^3^ Department of Medicine Yazd Branch, Islamic Azad University Yazd Iran

**Keywords:** MASI score, melasma, oral tranexamic acid, topical tranexamic acid, tranexamic acid

## Abstract

**Background:**

Melasma is a prevalent skin condition that primarily affects females of reproductive age. Despite the various available treatments, managing melasma is challenging due to frequent relapses and partial responses. Tranexamic acid (TXA) has gained attention as a potential treatment because of its antifibrinolytic and anti‐melanogenic properties. However, there is still limited information on the efficacy of oral versus topical TXA formulations.

**Aim:**

The present investigation explored the efficacy and safety of topical versus oral TXA in melasma patients to determine whether topical administration could provide a viable alternative with improved tolerability.

**Patients/Methods:**

In this single‐center randomized trial, 50 melasma patients received oral TXA (250 mg twice daily) or topical TXA (a 5% cream applied twice daily) for 12 weeks. Melasma severity was measured using the Melasma Area and Severity Index (MASI) score at baseline and after 3 months. Data analysis was performed using SPSS version 24, with *p* < 0.05 considered statistically significant.

**Results:**

Fifty female patients (mean age≈39.9 years) were equally randomized to the oral and topical TXA group. One patient in the topical group discontinued treatment due to sensitivity, while all in the oral group completed the study. At baseline, MASI scores were not significantly different between the two groups (*p* = 0.28). After 12 weeks, both groups demonstrated significant reductions in MASI scores (oral group: 58.86%; topical group: 50.88%; *p* = 0.001 for each), though the difference was insignificant.

**Discussion:**

Fifty female patients (mean age≈39.9 years) were equally randomized to the oral and topical TXA groups. One patient in the topical group discontinued treatment due to sensitivity, while all in the oral group completed the study. Baseline MASI scores were similar (*p* = 0.28). After 12 weeks, both groups demonstrated significant reductions in MASI scores (oral group: 58.86%; topical group: 50.88%; *p* = 0.001 for each), though the difference was not statistically significant.

**Conclusion:**

Both forms of TXA are effective with low side effects. The choice between oral and topical TXA can depend on patient preference and convenience.

## 1 Introduction

Melasma is a common acquired pigmentary skin condition characterized by dark patches or macules appearing on sun‐exposed areas of the face, like the forehead, malar regions, and chin, that usually affects females of reproductive age [1, 2]. Its prevalence ranges from 9% in the United States to 40% in Asia [3]. Melasma has an important effect on social function, emotional state, and the quality of life [4, 5].

Several factors have been incriminated in the pathogenesis of melasma including genetic influences, contraceptive pill, elevated levels of progesterone and estrogen during pregnancy, sun exposure, thyroid disease, malnutrition, liver dysfunction, use of cosmetic products, and drugs like tetracycline, minocycline, and Dilantin, leading to activation of melanocytes and producing excessive melanin. Histological changes include mast cells, increased melanocytes, solar elastosis, and vascular endothelial growth factor, along with a thinner basement membrane [6, 7, 8, 9].

In spite of the availability of various treatment options including chemical peels, topical depigmenting agents, procedural treatments, and energy‐based devices, the incomplete treatment and inadequate response highlight the need for more effective treatments [7, 10].

Tranexamic acid (TXA) is a common treatment of melasma that has antiplasmin properties. It is a synthetic lysine analog that has shown promise in treating melasma through several mechanisms. It primarily works by inhibiting plasmin formation, which blocks plasminogen activation and limits ultraviolet (UV)‐induced melanogenesis [11]. This inhibition is crucial in restricting UV‐induced melanogenesis. In response to UV radiation, increased plasmin activity in keratinocytes releases arachidonic acid, transforming it into prostaglandins that stimulate melanin production. By preventing this process, TXA indirectly reduces the activation of melanocytes. Additionally, TXA may reduce tyrosinase activity, likely due to its structural similarity to tyrosine [12, 13].

Given the various ways TXA may influence melanogenesis, its potential for treating melasma has garnered increasing interest. Although oral and topical formulations exist, it remains uncertain whether the topical route can achieve similar efficacy with enhanced safety. Therefore, this study evaluated and compared the therapeutic outcomes of topical versus oral TXA in patients with melasma.

## 2 Materials and Method

This single‐center, prospective randomized clinical trial study was carried out on patients attending a department of dermatology in Yazd, Iran, over 3 months (January to February 2021). The present investigation is approved by the institutional ethics committee (IR.SSU.MEDICINE.REC.1398.065), and retrospectively registered in the IRCT (IRCT20181208041882N9). Written informed consent was taken from all subjects to voluntarily participate. After a thorough clinical examination, patients who fulfilled the inclusion and exclusion criteria were enrolled.

The inclusion criteria were: women aged 18 to 60, with Fitzpatrick skin types III or IV, who exhibited features indicative of melasma and had a history of the condition for more than 6 months. The exclusion criteria were as follows: pregnancy, lactation, having major systemic diseases, suffering from coagulation disorders, not willing to follow up, and a history of taking drugs like oral contraceptives, anti‐pigmentations, photosensitizing, or melasma therapy within 6 months prior to the study.

In total, 50 out of 76 women with melasma were randomized into two groups based on a computer‐generated randomization sequence: one group received oral TXA, and the other used a topical formulation. The oral TXA group was prescribed 250 mg of TXA capsules (Amin Pharmaceutical Co., Iran) twice daily. Meanwhile, the topical TXA group was instructed to apply 5% topical TXA cream to the whole face twice daily. Both groups were instructed to apply one type of sunscreen (SPF 60, board‐spectrum, Cinere, Iran) in the morning at an interval of 3 h. Additionally, the usage of cosmetics or any other treatments was restricted.

The 5% TXA cream was specifically formulated for stability and efficacy. It consisted of 15 g of TXA powder (≥ 99%, Merk, Germany) mixed with 30 g of propylene glycol (≥ 99%, Merk, Germany) and 255 g of an O/W cream base (Cold cream, Farabi Pharmaceutical Co., Iran), resulting in a total batch weight of 300 g, which was packaged into 30‐g containers. The ingredients were accurately weighed using a digital scale (A&D company, Limited FX300‐GD). Since the cream is preservative‐free, its stability lasts only 30 to 40 days, requiring small batch production and prompt distribution. Patients received clear usage instructions and expiration details to ensure safe and practical application.

Before beginning the treatment protocol (baseline) and at the conclusion of 12 weeks, all patients were clinically examined by a single skilled dermatologist. The severity of melasma was measured using the Melasma Area and Severity Index (MASI) score devised by Kimbrough‐Green et al. [14]. The MASI score accounts for 30% of involvement in the forehead (F), 30% in both the left and right malar regions (LM and RM), and 10% in the chin (C). Three variables are used to determine the score:

1. Area involvement (A): This is assessed on a scale from 0 to 4, where: 0 = 0%; 1 = < 10%; 2 = 10% to 29%; 3 = 30% to 49%; 4 = 50% to 69%; 5 = 70% to 89%; and 6 = 90% to 100%.
2. Darkness of melasma (D): This is rated on a scale from 0 to 4: 0 = absent; 1 = slight; 2 = mild; 3 = marked; and 4 = severe.
3. Homogeneity of hyperpigmentation (H): This is assessed on a scale from 0 to 4: 0 = minimal; 1 = slight; 2 = mild; 3 = marked; and 4 = maximum.

In brief, MASI was calculated using the following formula:

$$\begin{array}{c} \text{MASI}=0/\text{3AF}\;\left(\mathrm{DF}+\mathrm{HF}\right)+0/\text{3ALM}\;\left(\mathrm{DLM}+\mathrm{HLM}\right)\\ \;+0/\text{3ARM}\;\left(\mathrm{DRM}+\mathrm{HRM}\right)+0/\text{1AC}\;\left(\mathrm{DC}+\mathrm{HC}\right) \end{array}$$

The range of the total score is 0 to 48. The scores calculated by a single‐blinded trained researcher to avoid bias result. In addition, patients were assessed every 2 weeks for any side effects of TXA including systemic side effects (nausea, diarrhea, abdominal pain, headache, dizziness, oligomenorrhea, hyposexuality, and thromboembolic events) and topical ones (erythema, pruritus, skin irritation, xerosis, and acne).

All data were registered in an accurate checklist. Values are expressed as mean ± standard deviation. The data were evaluated using *t*‐test, ANOVA, and chi‐square statistical tests and analyzed by SPSS version 24 for Windows (SPSS; Chicago, IL, USA). A confidence interval of 95% was used, with a *p*‐value of < 0.05 considered statistically significant.

## 3 Result

**FIGURE 1:**
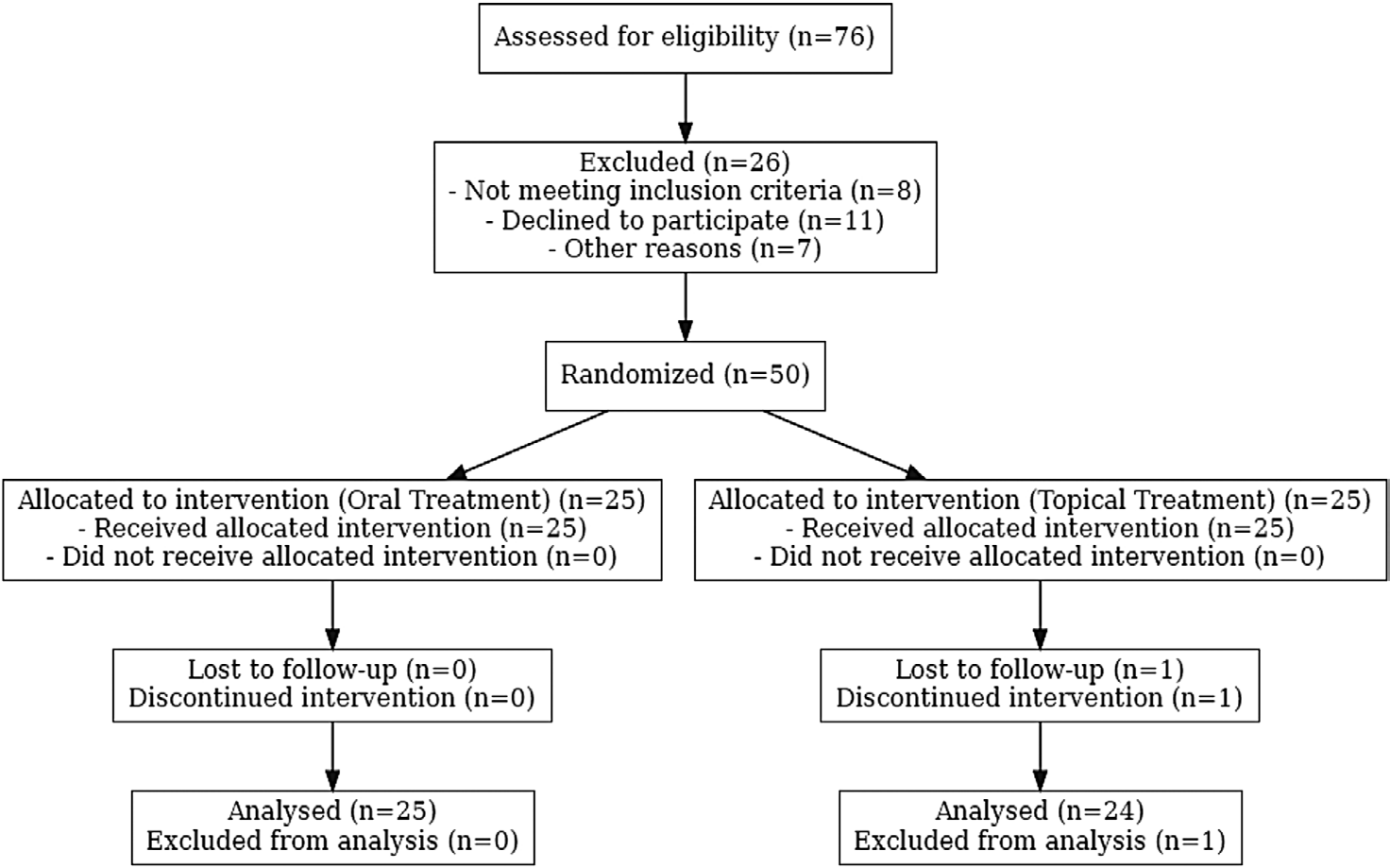
Details of the enrollment, allocation, and analysis of population.

Recruitment for this investigation began in January 2021 in Yazd, Iran. According to Figure 1, out of the 76 patients diagnosed with melasma by an experienced dermatologist, 26 were excluded from the study. Eight of these did not meet the inclusion or exclusion criteria: three were pregnant or breastfeeding, one had lupus, and four had recently used oral contraceptives. Additionally, 11 patients chose not to participate: five were concerned about potential side effects, three lost interest in following up, and three withdrew for personal reasons. Furthermore, seven other patients dropped out for other reasons, like difficulty managing the medication properly and having other skin conditions that could have interfered with the study results.

Ultimately, 50 patients were randomly assigned to either the oral TXA group (*n* = 25) or the topical treatment group (*n* = 25). The mean age (±SD) was 39.8 (±4.1) and 39.95 (±6.2) years in the topical and oral TXA groups, respectively (*p* = 0.92). Only one subject in the topical TXA group discontinued the 3‐month treatment due to drug sensitivity, which manifested as erythema and pruritus. Also, oligomenorrhea was reported by four patients (16%) of the oral TXA group; however, they voluntarily completed the course. No other reverse effects were observed.

**GRAPH 1:**
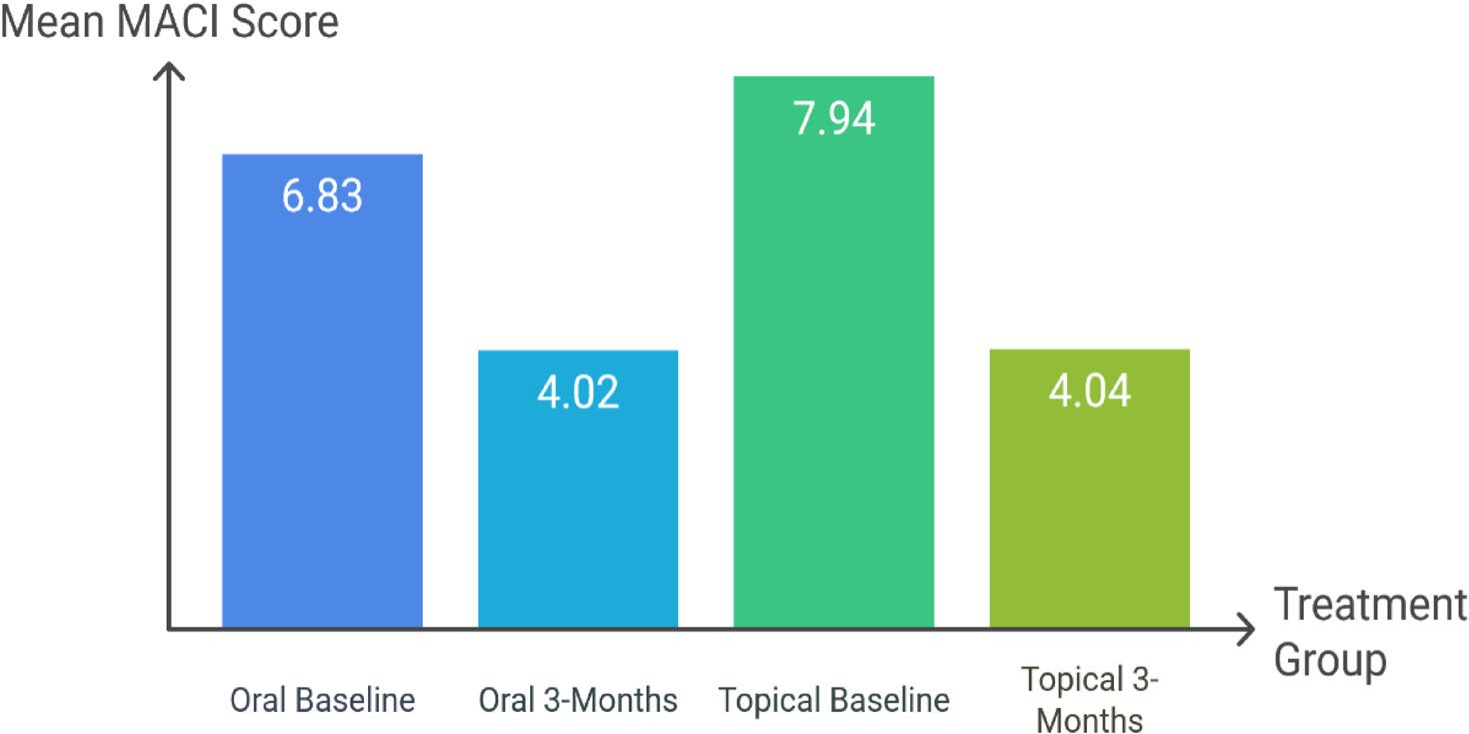
Mean MACI score changes in each group at baseline and after 3 months treatment. *p* value ≤ 0.05 is statistically significant; TXA, tranexamic acid.

According to Table 1, at baseline, no significant differences were exposed between the mean MACI score of the two groups (6.83 ± 3.84 in the oral TXA group and 7.94 ± 3.36 in topical TXA group, *p* = 0.28). After 3 months of administered TXA, all the enrolled patients showed a decrease in MASI score, and about 43% of them achieved a reduction of more than half of their initial MACI score. However, the difference between the mean MACI score of the two groups was not found to be statistically significant by the end of the period. There was a 58.86% decrease in mean MASI in the oral TXA group compared to a 50.88% reduction in the topical TXA group, which indicates statistically significant improvement in both oral and topical TXA groups (*p* = 0.001 in each group). Also, the significant changes in the MASI scores for both groups are presented in Graph 1.

**TABLE 1:** Comparison of MACI scores in both groups at baseline and after 3 months of treatment.

Measurement time	Type of administration	Mean MACI score	*p*
At baseline	Oral	6.83 ± 3.84	0.28
	Topical	7.94 ± 3.36	
After 3‐month treatment	Oral	4.02 ± 2.48	0.97
	Topical	4.04 ± 2.12	

*Note: p* ≤ 0.05 is statically significant.

## 4 Discussion

Melasma is a prevalent pigmentary skin disease, acquired symmetrically, characterized by the presence of dark patches and macules involving photoexposed areas of the face like the forehead, malar regions, and chin [1, 6, 12]. Multiple factors have been suspected to play a role in the development of melasma, including genetic influences, hormones, sun exposure, and vascular factors that result in melanocyte activation and increased melanization [7, 8, 9].

Among various treatments of melasma, TXA seems to be an effective and safe drug [15]. Recently, there has been increasing interest in examining the impacts of TXA on melasma [6]. Research indicates that the oral use of TXA for treating melasma is safe for up to 6 months, making it a promising option for individuals seeking relief from this skin condition [16]. In this study, a comparison was made between oral and topical TXA over a duration of 3 months (Table 2).

**TABLE 2:** Comparison of MACI score changes within each group separately at baseline and after 3 months of treatment.

Study groups	Mean MACI score at baseline	Mean MACI score after 3‐months treatment	*p*
Oral	6.83 ± 3.84	4.02 ± 2.48	0.001
Topical	7.94 ± 3.36	4.04 ± 2.12	0.001

*Note: p* ≤ 0.05 is statically significant.

In the 49 individuals who completed the study, the baseline MASI score was 7.40, ranged from 1.20 to 15.60. This score in studies conducted by Prathyoosha et al. [7], Del Rosario et al. [17] were 13.08 and 8.52 respectively. The cause of this diversity seems to be the various occupational sun exposures in different geographical regions.

In the oral TXA group, the findings indicated a significant decrease in the mean MASI score from a baseline of 6.83 to 4.02 after 12 weeks, which is consistent with other investigations by Prathyoosha et al. and Sheikh et al. [7, 15]. Also, the MASI score in the topical TXA group significantly decreased from 7.94 to 4.04 in accordance with the study conducted by Agrawal et al., who reported a remarkable improvement in MASI scores at 12 weeks [6]. In comparing the efficacy of two treatment routes, the difference in mean MASI scores after 12 weeks between the oral and topical TXA groups was not significant (*p* = 0.97). This result aligns with the study conducted by Devi et al. [18], but contrasts with Agrawal et al.'s findings [6]. This convergence in findings may be explained by variations in genetic backgrounds or skin characteristics across different populations.

In the current study, only one patient in the topical TXA group discontinued treatment due to drug sensitivity. Also, no significant number of serious side effects were reported by other studies, and adverse effects were transient and selflimiting [7, 17].

## 5 Conclusion

In this study, oral and topical TXA showed similar effectiveness in reducing melasma severity over a 12‐week period, with minimal adverse effects. Given the comparable clinical results and the added convenience of topical application, topical TXA may represent a suitable alternative in routine practice. These findings highlight the need for further investigations across different populations to clarify the influence of genetic and environmental factors on treatment response.

## 6 Limitation

Patients with severe melasma may require a longer course of therapy, and further investigation into this possibility is needed. The study has a few limitations, including a small sample size, a non‐blinded design, and the lack of dermoscopic evaluation. Furthermore, for cases of resistant and persistent melasma, longer treatment periods and extended follow‐up would be necessary.

## Author Contributions

Conceptualization and writing: Fateme Heidari; formal analysis: Behrooz Heidary; supervision, Vahid Ramezani; physical examiner: Mohammad Ebrahimzadeh Ardakani; data collecting, Golnaz Alsadat Habibzadeh Meibodi. All authors have read and agreed to the published version of the manuscript.

## Ethics Statement

The authors confirm adherence to the journal's ethical policies and have received approval from the Shahid Sadoughi Medical University Ethics Committee (IR.SSU.MEDICINE.REC.1398.065). Additionally, the study retrospectively registered in the IRCT (IRCT20181208041882N9).

## Consent

Informed consent was obtained from all patients involved in this study.

## Conflicts of Interest

The authors declare no conflicts of interest.

## Data Availability

The data that support the findings of this study are available on request from the corresponding author. The data are not publicly available due to privacy or ethical restrictions.
