# Correction to “Randomized Clinical Trial on the Efficacy of Oral Tranexamic Acid Versus Topical Tranexamic Acid in Treatment of Melasma”

**DOI:** 10.1111/jocd.70516

**Published:** 2025-11-21

B. Heidary , G. A. H. Meibodi , M. E. Ardakani , V. Ramezani , F. Heidari , “Randomized Clinical Trial on the Efficacy of Oral Tranexamic Acid Versus Topical Tranexamic Acid in Treatment of Melasma,” Journal of Cosmetic Dermatology 24, no. 9 (2025): e70428. 10.1111/jocd.70428 40923777 PMC12418907

Description of error:

In the originally published version of the manuscript, the name and affiliation of the first author are incorrectly listed as:

Name: Behrooz Heidary

Affiliation: Department of Pharmaceutics, Faculty of Pharmacy, Shahid Sadoughi University of Medical Sciences, Yazd, Iran

The correct form should be:

Name: Behrooz Heydari

Affiliation: Department of Clinical Pharmacy, Faculty of Pharmacy, Shahid Sadoughi University of Medical Sciences, Yazd, Iran

We apologize for this error.

